# Phase-matched five-wave mixing in zinc oxide microwire

**DOI:** 10.1515/nanoph-2024-0129

**Published:** 2024-07-24

**Authors:** Kaibo Cui, Tianzhu Zhang, Tao Rao, Xianghui Zhang, Shunping Zhang, Hongxing Xu

**Affiliations:** School of Physics and Technology and Key Laboratory of Artificial Micro- and Nano-structures of Ministry of Education, Wuhan University 12390 , Wuhan 430072, China; Hubei Key Laboratory of Micro-Nanoelectronic Materials and Devices, School of Microelectronics, Hubei University, Wuhan 430062, China; Wuhan Institute of Quantum Technology, Wuhan 430206, China; School of Microelectronics, Wuhan University 12390 , Wuhan 430072, China; Henan Academy of Sciences, Zhengzhou 450046, Henan, China

**Keywords:** nonlinear nanophotonics, ZnO microwire, phase matching, five-wave mixing, sum frequency generation

## Abstract

High-order wave mixing in solid-state platforms gather increasing importance due to the development of advanced lasers and integrated photonic circuit for both classical and quantum information. However, the high-order wave mixing is generally inefficient in solids under weak pump. Here, we observed the presence of phase matching of five-wave mixing (5WM) propagating in a zinc oxide (ZnO) microwire. The 5WM signal is enhanced by 2–3 orders of magnitude under the phase matching conditions, reaching an absolute conversion efficiency of 1.7 × 10^−13^ when the peak pumping power density is about 10^6^ W/cm^2^. The propagation of multiple nonlinear signals, including sum frequency generation, third harmonic generation, four-wave mixing etc., benefited from both the large nonlinear coefficients and the wide transparent window of ZnO, implies the possibility of developing cascaded nonlinear process under higher pumping. This study enriches the ZnO platform for integrated nonlinear nanophotonics.

## 1 Introduction

As a fundamental nonlinear optical process, wave mixing is important in modern optics and photonics, such as frequency conversion [1], [2], optical frequency comb [3], [4], quantum light source [5], [6], and all-optical modulation [7]. Since the wave mixing processes involve multiple photons, the degrees of freedom that can be controlled is related with the number of photons [8], meaning that the higher-order wave mixing takes a natural advantage in, e.g., broadening the accessible spectrum and generating more quantum-correlated photon pairs [9], compared to the lower-order ones. However, the efficiency of the high-order wave mixing is usually extremely low under the weak pump. A straightforward way to improve the efficiency is to increase the excitation power or to use artificial microstructures with giant field enhancement [10], [11], [12], [13], [14], [15], [16]. Due to the strong absorption in solids and the low damage threshold, efficient high-order wave mixing is generally demonstrated only in gas phase [17], [18] or at microwave frequencies [19], which cannot meet the demand of photonic integrated devices. To circumvent the obstacle, cascading multiple lower-order processes is one solution, but it needs an elaborately designed multiple resonant cavity and efficient lower-order process [20].

As a wide bandgap semiconductor, ZnO has a transparent window from visible to infrared [21]. The excitonic effect and hexagonal wurtzite crystal structure make it an ideal platform for integrated nonlinear devices, such as the second harmonic generation (SHG), third harmonic generation (THG) [22], [23], [24], [25], and high harmonic generation [26]. Recently, our group reported the extraordinary five-wave mixing (5WM) in ZnO crystals, attributed to the cooperative effect between the photonic resonances of the structure and some electronic energy levels in the crystals [27]. This finding indicates that ZnO micro- or nanowires can be a good material platform for developing high-order wave mixing. Extending the interaction length between the excitation and nonlinear materials is one straightforward way of increasing the nonlinear efficiency. Then, phase matching or quasi-phase matching becomes a critical requirement to ensure the coherent adding up of the generated nonlinear signals, as being widely investigated in other nonlinear optic platforms, such as SHG in lithium niobite waveguide [28], [29], [30], [31], [32], [33], [34], organic waveguide [35], GaAs [36], Si_3_N_4_ [37], [38], transition metal dichalcogenides [39], [40], etc. However, demonstration of phase-matched 5WM in solid-state platform remains elusive.

Here, we have experimentally studied the propagation of nonlinear signals in a *c*-axis ZnO microwire (MW) waveguide, pumped through a polarization-maintaining single mode fiber with a microlens. When the phase matching condition is satisfied, the 5WM intensity can be improved by 2–3 orders of magnitude. The simulation results indicate that the phase matching may originate from the nonlinear interaction between one lower mode at the pumping frequencies and one of the high-order modes at the signal frequency. Our study demonstrates that crystalline ZnO microstructure as an appealing material platform for efficient high-order wave maxing and it can in principle been hybrid integrated with other photonic platforms by various transfer techniques.

## 2 Materials and methods

**Figure 1:**
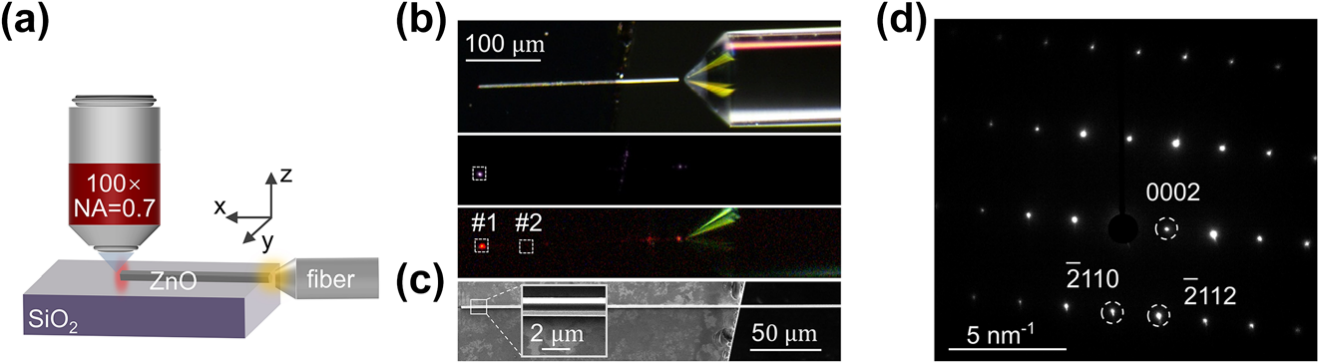
Measurement of nonlinear signals. (a) Schematic of a bare ZnO MW on SiO_2_ excited by optical fiber from the suspended end. The signal light is collected from the output end with a 100× objective lens. (b) From top to bottom, optical dark field image, near infrared light (1,220 nm) propagation imaging, and nonlinear signal imaging. The white dash boxes #1 and #2 indicate the collection region at the output end of the fiber and at the middle of the ZnO waveguide. Note that the actual collection area is smaller than the white boxes. The scale bar is 100 µm, and it applies to all the panels. (c) Scanning electron microscope image of a single ZnO MW on SiO_2_, one end hanging. The inset shows a magnified view of the main figure. (d) Selected area electron diffraction of a typical ZnO MW.

We choose ZnO MW other than nanowires because the diameter of the waveguide should be roughly larger than ∼800 nm to support multiple photonic modes at the pumping and signal frequencies. It also facilitates the excitation of waveguide modes in the experiment. The ZnO MWs were grown on the commercial sapphire substrate in a horizontal tube furnace by chemical vapor deposition method [41] and were dropped onto the substrate after being dispersed in ethanol. Under the optical microscope, we used a tungsten needle to pull a ZnO MW to the edge of the substrate so that one end is suspended in order to facilitate the end-fire coupling to the MW. A microlens (Raysung Photonics Inc., PM1550 nm-HP) at the end of the fiber is used to focus the pump laser onto the end facet of the ZnO MW at a distance of approximately 6.9 μm. The cone angle of the fiber is 90 ± 5°, which focus light into a spot with a beam waist of 2 ± 0.5 μm. Near infrared pulse (1,080–1,580 nm, repetition rate 20 MHz and pulse duration of 100 ps) was picked by acoustic optic tunable filter from a supercontinuum laser (YSL photonics-SC PRO), with a typical spectral width of 6 nm. As shown in Figure 1(a), ZnO waveguide was excited by the lensed fiber, and the signal light propagating to the output end is collected by a 100× objective (Mitutoyo M Plan Apo NIR, NA = 0.7). The three images in Figure 1(b) were taken by the same CCD (Nikon DS-Ri1). The top one is a dark-field image of the ZnO and fiber. The middle and lower pictures correspond to the imaging at the pump light (1,220 nm) and nonlinear signal images, filtered by a dichroic mirror (Semrock FF875-Di01). From the middle image, the intensity of the light at the output terminal is obviously larger than that at connection region between the microlens and the ZnO waveguide. This implies that most of the infrared pumping pulse is well coupled into the ZnO waveguide. Figure 1(c) shows the scanning electron microscope image of the ZnO MW used in the experiment. The length of the ZnO MW is 270 μm, and the width is 1.8 μm, slightly smaller than the beam spot focused by the microlens. Selected area electron diffraction in Figure 1(d) displays the representative diffraction pattern of hexagonal wurtzite of the ZnO nanowire.

## 3 Results and discussion

**Figure 2:**
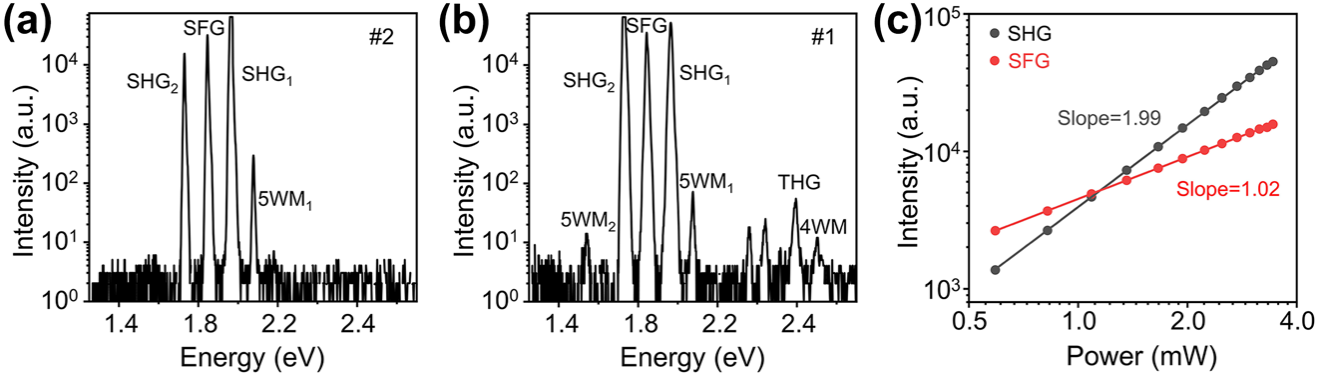
Representative nonlinear spectrum. (a) Nonlinear spectra at ZnO MW excitation position under normal incident excitation, *ℏω*_1_ = 0.984 eV and *ℏω*_2_ = 0.867 eV. (b) Nonlinear spectra at ZnO MW output terminal position under fiber coupling, *ℏω*_1_ = 0.984 eV and *ℏω*_2_ = 0.867 eV. (c) Logarithmic power dependence of the SHG_2_ (black dots) and SFG (red dots) on excitation 1 from (b), when the power of excitation 2 is fixed. Linear fittings yield slopes of 1.99 and 1.02.

In order to identify the observed nonlinear process, we first collected the signals at the middle of ZnO MW (region #2), pumped by a normal incidence laser from the objective lens (Figure 2(a)). All measurements were taken at room temperature. Two laser beams, excitation 1 (*ℏω*_1_) and excitation 2 (*ℏω*_2_), propagate collinearly and focus onto the ZnO MW. When the photon energies of the two laser beams are 0.984 eV (*ℏω*_1_) and 0.867 eV (*ℏω*_2_), there are four peaks in the spectrum (Figure 2(a)), three of which (1.730, 1.968, 1.846 eV) correspond to SHG from excitation 1 (2*ω*_1_), SHG from excitation 2 (2*ω*_2_), and sum frequency generation (SFG, *ω*_1_ + *ω*_2_). The rest signal at 2.077 eV is 5WM produced also by the synergistic interaction of the two laser beams, corresponding to 3*ℏω*_1_ − *ℏω*_2_, as we will show later. Figure 2(b) shows the nonlinear spectrum at the ZnO MW output terminal (region #1), pumped by two laser pulses of the same wavelengths as in Figure 2(a), but from the fiber coupling. Similar to the spectrum taken at region #2, the SHG, SFG, and 5WM signals can also propagate along the ZnO waveguide to the output end. The difference between the two nonlinear spectra in Figure 2(b) exhibits extra peaks from third-order nonlinear processes, such as THG and four-wave mixing (4WM). Figure 2(c) depicts the power dependence of SHG and SFG in Figure 2(b), by fixing the power of excitation 2 at 3.2 mW and changing the power of excitation 1 from 0.2 mW to 3.4 mW. The fit slopes of 1.99 and 1.02 are close to the expected quadratic and linear dependence.

**Figure 3:**
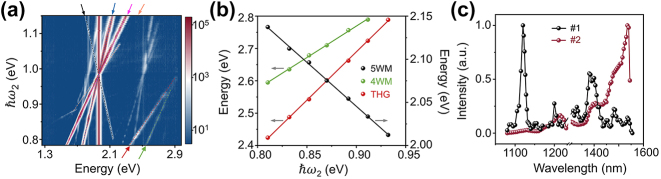
Nonlinear spectrum with variable wavelength. (a) Color map of the nonlinear signal intensity. The pink, red, blue, and green arrows represent the SHG, THG from excitation 2, SFG, and 4WM, respectively. The black and orange arrows show 5WM with *ℏω* = 3*ℏω*_1_ − *ℏω*_2_ and *ℏω* = 3*ℏω*_2_ − *ℏω*_1_, respectively. (b) Slope fitting of the nonlinear signal corresponding to the black, red, and green arrows in (a). (c) Normalized intensity value of 5WM. The black and red dots correspond to 5WM at the output terminal (#1) of the fiber coupling and 5WM at the excitation position (#2) of the normal incident objective excitation, respectively.

To confirm the 5WM signal, the wavelength of excitation 2 was scanned from 0.785 eV to 1.148 eV, with the *ℏω*_1_ fixed at 0.984 eV (1,260 nm) and a power of 1.74 mW (peak power 0.87 W). Consider a pump spot width of 2 μm from the fiber lens, the peak power density at the ZnO end facet is 6.7 × 10^6^/W/cm^2^. Figure 3(a) shows the color map of the nonlinear intensity as a function of *ℏω*_2_. Figure 3(b) shows the variation of the peak position as a function of *ℏω*_2_. Linear fittings to these energy dependence show that the slopes for the 5WM, 4WM, and THG are −1.01, 1.94, and 2.98, with intercepts of 2.96, 1.02, and 0.003. For 5WM, the slope and intercept are close to −1.0 and 3.0, in well accordance to *ℏω* = 3*ℏω*_1_ − *ℏω*_2_, implying an annihilation of three photons and the creation of one idle photon and one signal photon. This fitting intercept of 2.96 is very close to 3*ℏω*_1_ (2.952 eV), further conforming the consistency. For 4WM (2*ℏω*_2_ + *ℏω*_1_) and THG (3*ℏω*_2_), the slopes of 1.94 and 2.98 are also close to the integers of 2 and 3. The intercepts of 1.02 and 0.003 are close to *ℏω*_1_ (0.984 eV) and 0, as expected.

Due to the dispersion of the ZnO material, the effective refractive indexes of waveguide modes at different frequencies are different. The pumping pulses and the generated nonlinear signal, in form of different waveguide modes, experience wave vectors mismatch during the propagation. The nonlinear coupled-mode theory can be used to describe the process [42]. For 5WM, the intensity change during propagation can be given by the following formula,

(1) $$A_{3}=\frac{i\omega_{5\text{WM}}}{4}\kappa A_{1}^{3}A_{2}^{*}x\cdot \text{sinc}\left(\frac{\Delta kx}{2}\right)$$

where *A*_3_ is the field amplitude of 5WM, *A*_1_ and *A*_2_ represents the amplitudes of the two excitation fields, *κ* is the modal overlap factor, which represent the nonlinear interaction strength between the pumping frequencies and the signal frequency. Δ*k* = 3*k*_1_ − *k*_2_ − *k*_3_ is the wavevector mismatch. When phase matching condition (Δ*k* = 0) is satisfied, the intensity of 5WM can be increased dramatically by orders of magnitude and depends quadratically on the interaction length *x*.

To illustrate the effect of phase matching, the wavelength-dependence 5WM signal collected at the output end (region #1) is compared with that in situ (region #2), as a function of the energy of excitation 2 (Figure 3(c)). Since the pumping power is not a constant when changing excitation 2 [peak power 0.62 (1,580 nm) ∼1.22 (1,350 nm) W], the intensity of 5WM is divided by the power of excitation 2. The propagated 5WM intensity values exhibit multiple extrema at about 1,120 nm, 1,200 nm, 1,332 nm, 1,381 nm, 1,400 nm, and 1,481 nm, with a maximum value at 1,120 nm. There is a sharp decrease in intensity on both sides of the maximum value, accompanied by slight oscillations and the maximum value is 2–3 orders of magnitude larger than the average value away from the maximum. The 5WM intensity at region #2 increases gradually with wavelength, reaching a maximum close to the boundary of the measurement range. Such spectral behavior was also observed in the ZnO microrod, independent on the aspect ratio of the microrods. But the wavelength-integrated 5WM intensity shows an enhancement when the aspect ratio is about 6 (see Section 1 in Supplementary Material). Due to the inconsistence in the position of the maximum 5WM intensity between the propagated and original locations, we can infer that this phenomenon is not caused by the propagation of 5WM generated at the excitation position to the output terminal, but rather represents a clear phase matching feature. Consider the coupling loss and 5WM emission efficiency, obtained by numerical calculation, the conversion efficiency can be calculated by *η*_5WM_ = *P*_5WM_/*P*_1_ *ξ*_in_ *ξ*_out_, where *P*_5wm_ (*P*_1_) is the power of 5WM (excitation 1, *ω*_1_), *ξ*_in_ denotes the coupling efficiency of excitation 1, and *ξ*_out_ denotes the emission efficiency of 5WM. The lower limit of the absolute conversion efficiency is 1.7 × 10^−13^ (see Section 2 in Supplementary Material). In the range from 1,235 nm to 1,305 nm, the peak of 5WM overlaps with the SHG and SFG. Since the intensity of 5WM was much smaller than that of SHG and SFG, it is covered by second-order nonlinear signals.

**Figure 4:**
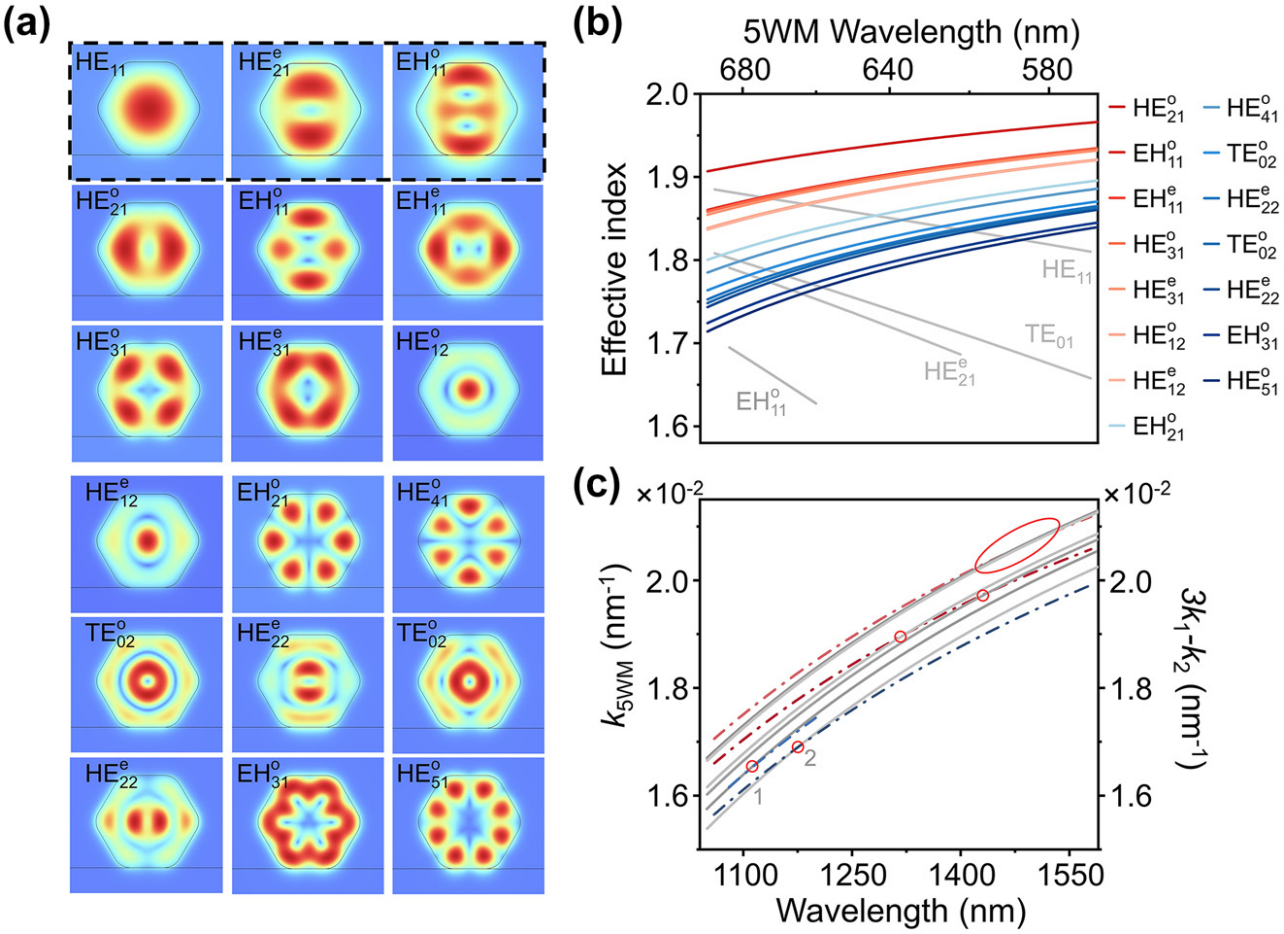
Dispersion curves for different guided modes. (a) Numerical simulations of normalized electric field amplitude distribution for the different modes in the ZnO waveguide. The wavelength (excitation 2) of the modes surrounded in black dashed boxes is 1,120 nm (1.107 eV), and the wavelength of the remaining modes is 672 nm (1.845 eV). (b) Effective refractive indexes of the guided modes as a function of the wavelength of excitation 2 and 5WM. The bottom axis is the excitation wavelength 2, corresponding to the four gray curves, and the upper axis is the 5WM wavelength obtained by *ℏω* = 3*ℏω*_1_ − *ℏω*_2_, with *ω*_1_ = 0.984 eV (1,260 nm), corresponding to the rest curves. (c) *k*_5WM_ (gray solid) and 3*k*_1_ − *k*_2_ (color dash dot) as a function of wavelength. The wavelength of *k*_1_ is fixed at 1,260 nm, each mode has a definite value for *k*_1_. The red circles mark the intersection between the curves.

To illustrate the phenomenon of phase matching in the experiment, two-dimensional mode analysis of the individual ZnO waveguide was conducted by using the finite element method (COMSOL Multiphysics V5.2a). The ZnO waveguide has a hexagonal cross section with a diameter of 1.8 μm and a rounded corner radius of 300 nm. The refractive indices of ZnO range from 1.94 (1,551 nm) to 2.03 (556 nm) for *n*_e_ (*c*-axis) and from 1.92 (1,551 nm) to 2.01 (556 nm) for *n*_o_, interpreted from Ref. [43]. Due to the larger size of the ZnO MW, it can support multiple modes at pumping frequency and 5WM frequency. Figure 4(a) displays all the modes in which phase matching is possible within the experimental measurement range, showcasing the modes akin to circular waveguides. These modes are named according to the nomenclature of circular waveguides [44], in which HE_11_ is the fundamental mode and the superscript e/o indicates the *x*-component of electric field being symmetric (even) or antisymmetric (odd) about the *x*-axis. HE (EH) means the modes have both magnetic and electric field components, but with the magnetic (electric) component dominates. For a given frequency, the higher-order modes always have lower effective refractive indices. Therefore, the condition of phase matching *k*_5WM_ = 3*k*_1_ − *k*_2_ cannot be achieved between a pair of identical modes since the material dispersion is larger in the visible range than in the near infrared for ZnO. However, the material dispersion can compensate for the mismatch in wave vectors if the high-order mode is at higher frequency. Figure 4(b) shows the real part of the effective refractive index for the guided modes at both the excitation and 5WM wavelength. All the effective refractive indexes decrease as the wavelength increases, due to the material dispersion. The dispersion curves show similar tendency since these modes are well confined in the ZnO WM whose *n*_e_ and *n*_o_ possess similar dispersion. In order to find the possible phase matching points, different combinations of modes were calculated and those combinations have intersections between 3*k*_1_ − *k*_2_ and *k*_5WM_ in the experimental wavelength range are shown in Figure 4(c). Around the experimental peak of 1,120 nm in Figure 3(c), the curves of *k*_5WM_ and 3*k*_1_ − *k*_2_ cross at 1,132 nm and 1,145 nm, marked by the circle 1 and circle 2. The corresponding waveguide modes at circle 1 are HE^e^_21_ (*k*_1_), EH^o^_11_ (*k*_2_), and HE^e^_22_ (*k*_5WM_) at circle 1, and TE_01_ (*k*_1_), TM_01_ (*k*_2_), and EH^o^_31_ (*k*_5WM_) at circle 2. There are other intersections at about 1,325 nm, 1,440 nm, 1,481 nm, and 1,525 nm, which illustrate multiple phase matching points in this waveguide. Due to the difference of actual experimental parameters from simulated geometric parameters, the numerical results show a slight deviation from the value measured in the experiment. Moreover, considering that the conversion efficiency relies on the spatial symmetry between the pumping frequency modes and 5WM modes, the variation in the intensity of different extrema of the 5WM in Figure 3(d) may be attributed to the modal overlap, if neglecting the variation of effective nonlinear susceptibility.

## 4 Conclusions

In summary, we have demonstrated the propagation of high-order wave-mixing process in solid by using near infrared pulse. In experiment, one end of the *c*-axis ZnO waveguide is suspend and excited by a microlens at the end of the polarization-maintaining single-mode fiber. When the fundamental wavelength is scanned, the phase matching condition is satisfied at specific wavelengths, at which the intensity of the 5WM increases by 2–3 orders of magnitude. The lower limit of the conversion efficiency is about 1.7 × 10^−13^. The multiple extremes of the propagating 5WM indicate that there are multiple phase matching points in the ZnO MW. Through the simulation calculation, we discuss the possible combination of waveguide modes that satisfies the phase matching conditions. The multiple intersection points between the curves of the *k*_5WM_ and 3*k*_1_ − *k*_2_ prove the existence of multiple phase matching points in the ZnO MW. Moreover, the concurrence of second-, third-, and fourth-order nonlinear processes are also observed, with a peak pumping power as low as 1 W. Our study provides a platform for high-order wave frequency mixing and coherent light sources, as well as for further exploring higher-order nonlinear processes and developing cascaded nonlinear process in solid [45]. More delicate designs of microstructure such as ridge waveguides, microrings, etc. can be fabricated on bulk ZnO crystal to further improve the conversion efficiency. But it should be noted that in ZnO crystal without defect-related photoluminescence, 5WM is quite weak [27]. Certain amounts of defects can be introduced into the bulk ZnO during the grown process or by ion implantation.

## Supplementary Material

Supplementary Material Details
