# Supplementary material for: Phase-matched five-wave mixing in zinc oxide microwire
Source: Nanophotonics. 2024 Jul 24;13(18):3403–9. doi: 10.1515/nanoph-2024-0129 (PMC11501356; doi:10.1515/nanoph-2024-0129)
Supplement: Supplementary file 1 — Supplementary Material Details [file j_nanoph-2024-0129_suppl_001.docx]

**Supplementary Material**

**Phase matched five-wave mixing in zinc oxide microwire**

Kaibo Cui^1^, Tianzhu Zhang^1^, Tao Rao^2^, Xianghui Zhang^2^, Shunping Zhang^1, 3, *^, Hongxing Xu^1, 3, 4, 5^

^1^School of Physics and Technology and Key Laboratory of Artificial Micro- and Nano-structures of Ministry of Education, Wuhan University, Wuhan 430072, China.

^2^Hubei Key Laboratory of Micro-Nanoelectronic Materials and Devices, School of Microelectronics, Hubei University, Wuhan 430062, China.

^3^Wuhan Institute of Quantum Technology, Wuhan 430206, China.

^4^School of Microelectronics, Wuhan University, Wuhan 430072, China.

^5^Henan Academy of Sciences, Zhengzhou 450046, Henan, China.

**^*^Corresponding author.** E-mail: [spzhang@whu.edu.cn](mailto:spzhang@whu.edu.cn).

S1: The 5WM spectra of different aspect ratio and diameter.

In order to know how the aspect ratio contributes to the 5WM output, we measured ZnO microrods of different length and diameter, as shown in Figure S1. Figure S1a shows the SEM image of a microrod. Figure S1b shows the polarization dependence of SHG excited by normal incident. For the measurement of 5WM, the orientation of the c-axis of all ZnO microrods is fixed, along the 0 degrees in Figure S1b. From Figure S1c, no obvious dependence of the spectra on the aspect of ZnO microrods is observed. But the wavelength integrated 5WM intensity shows an enhancement when the aspect ratio is about 6. For microrods with similar aspect ratio but different diameters, the 5WM signal is generally stronger for larger diameters (Figure S1d).


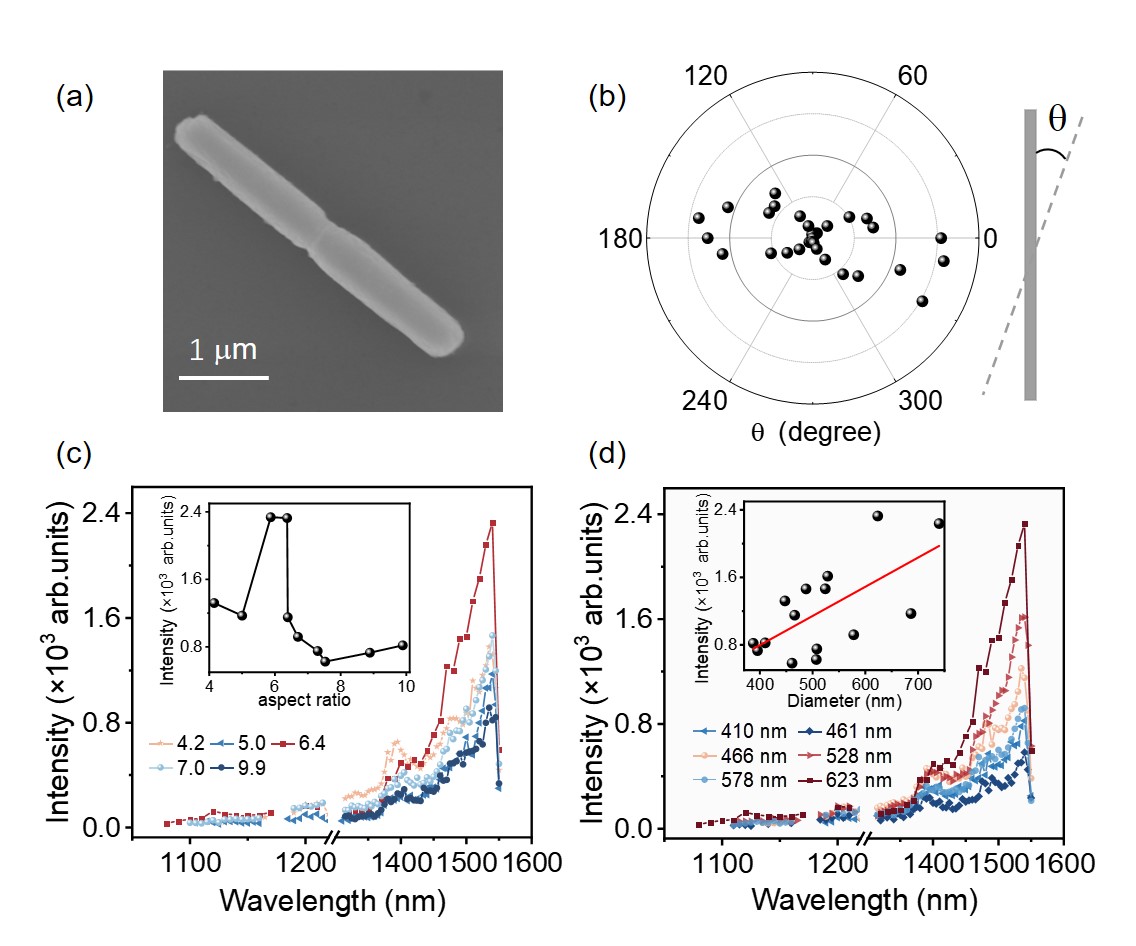


Figure S1 (a) SEM image of a ZnO microrod. (b) Polarimetric SHG of ZnO microrod, 0 degrees along the c-axis of ZnO microrods. (c, d) 5WM spectra taken from ZnO microrods of different aspect ratio (c) or diameter (d), the aspect ratio in (d) is fixed around 6.5. The insets for (c) and (d) show the intensity of 5WM varies with the aspect ratio and diameter at the fixed wavelength ( = 1260 nm,  = 1540 nm). Red line in the inset for guiding the eye. All the c-axis of ZnO microrods is along the polarization at 0 degrees (red point) in (b).

S2: simulation of the coupling and emission efficiency.

3D frequency domain finite element method was used to calculate the coupling efficiency. A Gaussian bema, with a beam waist radius of 1 μm, illuminate on the end facet of the ZnO (Figure S2a). By comparing the input intensity and the power propagated in the ZnO MW, we could obtain the coupling efficiency. At the range from 1100 nm to 1550 nm, the coupling efficiency is around 0.65 for different positions away from the coupling facet of ZnO MW (Figure S2b). The emission efficiency was calculated in a similar way (Figure S2c). Firstly, the 5WM mode is launched from the port and propagates to the output terminal, then by comparing the output intensity collected by an objective (NA = 0.7) with the input intensity, we can obtain the emission efficiency of different modes. The emission efficiency of different modes is quite different, ranging from 0.02 to 0.2 (EHO 11 0.065, HEe 12 0.0259, HEe 22 0.203, EHO 31 0.1223).

At the wavelength of phase matched 5WM in the main text, when the intensity of the laser is 1 w, the spectrometer integrated for 1s yields a reading around 2×10^10^. The power of the two pumping lasers *P*_1_ (  nm) and *P*_2_ (  nm) are 1.74 mW and 1.46 mW respectively. The reading of spectrometer integrated for 30s are 311. Therefore, we can estimate the output power of 5WM to be 3.94×10^-11^ W, the conversion efficiency can be expressed as

where *P*_5wm_ (*P*_1_) is the power of 5WM (excitation 1, *ω*__), _in_ denotes the coupling efficiency of* *excitation 1 and _out_ denotes the emission efficiency of 5WM. If we assume the coupling efficiency and the emission efficiency to be 0.65 and 0.2, the lower limit of the absolute conversion efficiency was about 1.7×10^-13^.


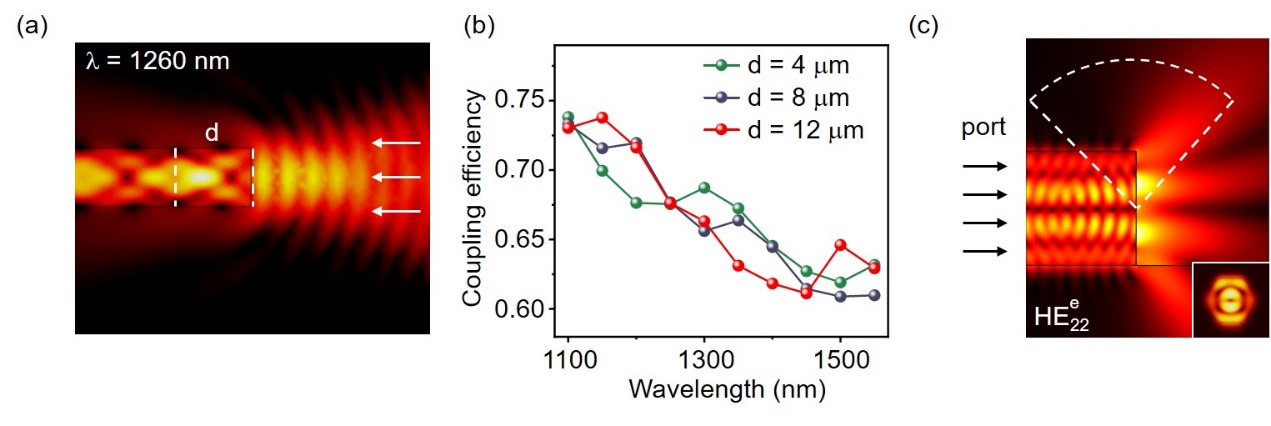


Figure S2. (a) Electric field distribution at the facet of ZnO MW illuminated by a Gaussian beam (beam width 2 μm) with wavelength of 1260 nm. White arrows show the direction of the Gaussian beam. The dash lines donate the cross section of ZnO, the distance between the integral cross-section and the ZnO end face is d. (b) The coupling efficiency varies with wavelength at three different positions away from the coupling face of ZnO MW. (c) Electric field distribution at the output terminal when the HEe 22 mode is launched at the left boundary of the ZnO MW (indicated by the black arrows). The white dash lines denote the collection angle corresponding to the N.A. of the objective used in the experiment.
